# The Influence of Maternal BMI on Adverse Pregnancy Outcomes in Older Women

**DOI:** 10.3390/nu12092838

**Published:** 2020-09-16

**Authors:** Małgorzata Lewandowska, Stefan Sajdak, Barbara Więckowska, Nevena Manevska, Jan Lubiński

**Affiliations:** 1 Medical Faculty, Lazarski University, 02-662 Warsaw, Poland; 2 Division of Gynecological Surgery, University Hospital, Poznan 60-535, Poland; ssajdak@ump.edu.pl; 3 Department of Computer Science and Statistics, Poznan University of Medical Sciences, 60-806 Poznan, Poland; barbara.wieckowska@ump.edu.pl; 4 Institute of Pathophysiology and Nuclear Medicine, University of Ss. Cyril and Methodius, 1000 Skopje, Macedonia; dr.nmanevska@gmail.com; 5 Department of Genetics and Pathology, International Hereditary Cancer Center, Pomeranian Medical University, 71-252 Szczecin, Poland; lubinski@pum.edu.pl

**Keywords:** maternal age, pregnancy results, hypertension, diabetes, newborn outcomes, obesity, underweight

## Abstract

As mothers age, the risk of adverse pregnancy outcomes may increase, but the results so far are controversial and several issues remain unknown, such as the impact of maternal weight on the effects associated with older age. In a prospective cohort of 912 Polish women with singleton pregnancies (recruited in 2015–2016), we assessed the pregnancy outcomes depending on the mother’s age (18–24, 25–29, 30–34, 35–39, and ≥40 years). Women aged ≥35 years (vs. <35 years) were assessed in terms of body mass index (BMI). Multidimensional logistic regression was used to calculate the odds ratios (with 95% confidence intervals) of the pregnancy results. The risk profiles (using the Lowess method) were applied to determine the threshold risk. We found that both the youngest and the oldest group members displayed higher adjusted odds ratios of preeclampsia (PE), intrauterine growth restriction (IUGR), and preterm birth <37th week (U-shaped risk). In the remaining cases, the age ≥40 years, compared to the youngest age 18–24 years, was associated with a higher adjusted risk of gestational hypertension (GH) (AOR = 5.76, *p* = 0.034), gestational diabetes mellitus GDM-1 (AOR = 7.06, *p* = 0.016), cesarean section (AOR = 6.97, *p* <0.001), and low birth weight LBW (AOR = 15.73, *p* = 0.033) as well as macrosomia >4000 g (AOR = 8.95, *p* = 0.048). We found that older age ≥35 years (vs. <35 years) was associated with higher adjusted odds ratios of all the pregnancy outcomes investigated. In obese women, these adverse older age related results were found to be more intense in GH study, as well as (though weaker) in birth <37th week study, small-for-gestational age birth weight (SGA), LBW, large-for-gestational age birth weight (LGA), and macrosomia. In overweight women, these adverse older age related results were found to be more intense in preterm birth study, as well as (though weaker) in SGA and LBW. In underweight women, adverse pregnancy outcomes related to older age were more intense in a study of cesarean section. At the same time, underweight was associated with reversal of some negative effects of older age (we found lower odds ratios of GDM-1 diabetes). The maternal threshold age above which the risk of GH, PE, GDM, caesarean section, and preterm birth increased was 33–34 years (lower than the threshold of 35 years assumed in the literature), and the threshold risk of IUGR, LBW, SGA, LGA, and macrosomia was 36–37 years. Main conclusions: Older maternal age was associated with a higher chance of all kinds of obstetric complications. Older women should particularly avoid obesity and overweight.

## 1. Introduction

There is a general tendency for the average age of pregnant women to increase, including those women who become pregnant for the first time. Advanced maternal age (AMA) is usually defined as age 35 or more for the mother at the time of delivery of her baby [1]. Studies in European and American populations have shown that the percentage of pregnant women >35 years old has increased 4–8 times over the last 30–40 years [2,3,4,5,6]. The question is whether older age is associated with worse pregnancy outcomes. Are the effects of older age exacerbated by maternal obesity during the obesity epidemic?

The aging of the body is a progressive, degenerative biological process that restricts various physiological functions over time. The mechanisms of aging remain unclear, and there are many theories regarding this issue (over 300) [7,8,9]. Theories related to the contribution of oxidative stress are the most popular. They include increased production of oxidants (free radicals or reactive oxygen species (ROS)), a simultaneous reduction in the efficiency of antioxidant systems, and a reduction in the ability to respond to oxidative stress (involving various signaling pathways and antioxidant genes), and ultimately, the accumulation of the products of oxidative damage of nucleotides, proteins and lipids in the cells. DNA damage, the involvement of multiple biomarkers and of genetic factors are also part of these theories [7,8,9].

One of the key elements in the pathogenesis of adverse pregnancy outcomes (such as hypertension, diabetes, premature birth, or fetal growth restriction) is increased oxidative stress. A higher risk of these pregnancy complications has been associated with lower levels of antioxidants [10,11,12,13,14,15,16]. This suggests that the oxidative stress associated with aging may increase the risk of pregnancy complications. However, the results of the research available in the literature are debatable and controversial. Many studies have shown risks of adverse pregnancy outcomes and complications increasing along with maternal age [17,18,19,20,21]. However, other studies have not found these associations [2,22,23]. The reasons for the discrepancies may be different research methodologies, different reference categories of maternal age, or the lack of adjustment for confounding factors. 

In addition, several issues remain unknown, such as the impact of abnormal maternal body mass index (BMI) on the effects associated with older age (>35 years). Literature provides a lot of examples that confirm relations between mothers’ obesity (or overweight) and a higher risk of macrosomic newborn and cesarean section, as well as preterm birth, preeclampsia, and fetal growth restriction [24,25,26]. At the same time, associations between obesity and placental dysfunction were found [24]. Obesity is associated with insulin resistance, increased oxidative stress, and inflammation [11,24,27]. The risk of obesity increases along with the age [20]. Being underweight may be directly related to the deficiency of nutrients with antioxidant properties. The question whether inappropriate weight of a mother (obesity, overweight, or underweight) increases older age related adverse outcomes is still open.

Another issue to be determined is the assessment of the threshold value of the maternal age in the development of pregnancy complications, which may change along with the changes of the age structure in the population of pregnant women.

The purpose of our study was a comprehensive and detailed analysis of the adjusted risk of multiple adverse pregnancy outcomes for maternal age, conducted in a collaborative prospective cohort of pregnant women. In particular, our aim was to evaluate the odds ratios of adverse pregnancy outcomes and complications for different maternal age categories and for different reference age categories in order to be able to, inter alia, identify U-shaped effects (when the lowest and highest maternal age increases the risk). We have established a maternal age for the threshold risk of each pregnancy outcome. In addition, we assessed maternal age-related effects separately in the subgroup of obese, overweight, and underweight women.

## 2. Materials and Methods 

The current study was an analysis of data collected in a prospective cohort of pregnant women recruited at the Gynecology and Obstetrics Teaching Hospital of the University of Poznań, Poland, in 2015–2016. This hospital is a tertiary reference center. Recruitment and research were carried out in accordance with the standards of the Helsinki Declaration and the Bioethics Committee at the Medical University of Poznań (this committee expressed its consent, in the document no. 769/15). All the participants were informed about the purpose of the study and its course and signed the Informed Consent Form before completing the personal questionnaire.

### 2.1. Participants and Methods

The recruitment was carried out at the hospital’s Central Laboratory Testing Point, to which the women willing to perform any laboratory tests report voluntarily. Information about the recruitment for the study was available to everyone. Caucasian women from the Wielkopolska region were recruited. They were 18–45 years old at conception, had a singleton pregnancy, and their gestational age was 10–14 weeks. One condition for accepting the candidates was the absence of chronic diseases such as hypertension or diabetes, diseases related to kidney and/or liver dysfunction, as well as the absence of any immunological diseases/inflammatory diseases and thromboembolism. Another condition for inclusion was the absence of fetal defects and the delivery of a (phenotypically) normal child with a gestational age of ≥25 weeks.

During recruitment, the women provided their socio-demographic data and information about their pregnancy, obstetric history, general health history, and the family health history, as well as the information about the vitamin and microelement preparations and stimulants used. All this information was recorded in the Personal Questionnaire, which was filled in by the pregnant women themselves (in the presence of a trained midwife). All the women reported not using alcoholic beverages while pregnant.

At the next stage, after the delivery date, information on the course of pregnancy and delivery as well as the results of the newborn and information on the mother’s health was taken from the medical records.

The 1300 women who met the recruitment criteria expressed their willingness to participate in the study over a 12-month period in 2015–2016, and all of them were invited to participate in the study. After the end of pregnancy, 48 women were excluded due to various reasons including miscarriage before the 20th week of pregnancy, delivery before the 25th week, diagnosis of the child’s defect, hospitalization in due to severe infection during pregnancy, or thromboembolism, diagnosis of arterial hypertension before the 20th week or diabetes before the 18th week of pregnancy. Some were also excluded due to the lack of cooperation. We obtained incomplete data from another 340 women, which was also a reason for exclusion.

Ultimately, the cohort for which the data were analyzed included 912 women.

In the current analysis, we assessed the following maternal outcomes (obtained from the medical records): pregnancy induced hypertension including preeclampsia (PE, *n* = 24 (2.6%)) and gestational hypertension (GH, *n* = 113 (12.4%)), gestational diabetes mellitus treated with a diet (GDM-1, *n* = 125 (13.7%)) and treated with insulin (GDM-2, *n* = 21 (2.3%)), and cesarean section (*n* = 382 (41.9%)) regardless of the reason. 

The following adverse outcomes of the newborn were assessed: preterm birth <37th week regardless of the reason and method of pregnancy completion (*n* = 65 (7.1%)), intrauterine growth restriction (IUGR) (*n* = 21 (2.3%)), small-for-gestational age birth weight <10th percentile (SGA, *n* = 72 (7.9%)), large-for-gestational age birth weight >90th percentile (LGA, *n* = 99 (10.9%)), low birth weight <2500 g (LBW, *n* = 60 (6.6%)), and macrosomia >4000 g (*n* = 97 (10.6%)). 

Pregnancy-induced hypertension was defined as systolic blood pressure ≥140 mmHg and diastolic blood pressure ≥90 mmHg obtained in at least two measurements 4 hours apart, developing de novo after the 20th week of gestation (measured with an oscillometric apparatus in a sitting position). The criteria for the diagnosis of preeclampsia (PE) included de novo development hypertension as well as de novo development of one of the organ disorders such as renal dysfunction, thrombocytopenia, hepatic dysfunction, cerebral or visual symptoms, and pulmonary edema (in all the PE cases in this cohort only proteinuria ≥0.3 g/L was found). Gestational hypertension (GH) was diagnosed in the case of isolated hypertension [12].

Gestational diabetes mellitus (GDM) was diagnosed on the basis of an (2-h) oral glucose tolerance test (75 g) at 24–28 weeks of gestation [16].

Gestational age was assessed on the basis of an ultrasound examination. Intrauterine growth restriction (IUGR) was diagnosed based on an ultrasound examination as well. 

Newborn weight (in grams) was measured conventionally immediately after delivery using an automatic device. Neonatal SGA and LGA identification (<10th and >90th percentile) was performed on the basis of gender and gestational age percentile grids in the population from the region covered by the study [13].

### 2.2. Studied Variables

In the current study, the main independent variable was maternal age, and the aim of the study was to evaluate the effect of maternal age on neonatal outcomes and maternal complications. Maternal age was defined as the completed age in years at conception. The following age categories have been established: 18–24, 25–29, 30–34, 35–39, and ≥40 years, as well as ≥35 and <35 years. In subsequent analyses, the following reference groups were adopted: 25–29 years of age, 18–24 years of age, and <35 years of age.

The effect of maternal body mass index (BMI) on pregnancy results was also assessed and the whole cohort was divided into BMI categories. BMI was calculated as the quotient of the weight before pregnancy (self-reported) and height (in meters) squared, and BMI categories were adopted according to the recommendations of the World Health Organization: <18.5, 18.5–24.9, 25.0–29.9, and ≥30 kg/m^2^ for underweight, normal weight, overweight, and obesity, respectively. Maternal height (in cm) were taken from medical records. The normal BMI was a reference category.

Many variables (risk factors of adverse pregnancy outcomes) were reported, including pre-pregnancy BMI, gestational weight gain beyond the Institute of Medicine (IOM) recommendation, primiparity, smoking in the first trimester, hypertension in previous pregnancies, treatment of infertility, family history of chronic hypertension, diabetes in previous pregnancies, family history of diabetes mellitus, PE and GDM in the current pregnancy, fetal sex, premature rupture of membranes (PROM) cases, maternal height, and gestational age at birth, as well as education levels and financial status. Confounding variables were established separately for each Adverse Pregnancy Outcome (as below, in Statistical section). 

Gestational weight gain (GWG) was defined as the difference between the weight before delivery and the pre-pregnancy weight, and the GWG norms for each pre-pregnancy BMI category were adopted according to the 2009 Institute of Medicine (IOM) recommendations. Maternal weight (in kg) before pregnancy was taken from medical records.

The characteristics of the participants also included the assessment of the education level; lower education being synonymous with the education period of less than 12 years. Financial status was assessed on a 5-point Lickert scale, considering the answer to the question "is your household’s financial status sufficient for your needs"; lower financial status included such answers as “definitely No”, “rather No”, and “hard to say”.

### 2.3. Statistical Analyses

All the calculations were made using PQStat v1.8.0 software (Poznań, Poland). *p*-value lower than 0.05 was treated as statistically significant. In order to describe continuous variables medians (with interquartile ranges 25–75%, IQ) and to describe categorical variables frequency and percentages were given. For continuous variables the comparison between two independent groups of measurements was performed by the Mann–Whitney U test (normality assumption was not met); for categorical variables with ordered categories by the Cochran–Armitage test for trend and for binomial categories the Pearson chi-square test (or Fisher exact test when Cochran assumption was not met) were used. Normality assumption was tested by the Shapiro–Wilk test.

Statistical analysis of the assessment of the possible impact of age on risk of adverse pregnancy outcomes and complications was carried out on the basis of a logistic regression model for maternal age categories, compared to reference categories: 18–24 years or 25–29 years or <35 years. First, a one-dimensional model was built and as an effect size the raw odds ratio (OR) with a 95% confidence interval was given. Then, in the multidimensional model, the obtained effect size was corrected by providing adjusted odds ratio (AOR) along with a 95% confidence interval.

In the multidimensional analyses, corrections were made by several models: (a) primiparity, pre-pregnancy BMI, gestational weight gain beyond the IOM recommendation and smoking in the first trimester; (b) model—(a) plus hypertension in previous pregnancies, treatment of infertility and family history of chronic hypertension (for PE and GH); (c) model—(a) plus diabetes in previous pregnancies and family history of diabetes mellitus (for GDM); (d) model—(a) plus PE and GDM in the current pregnancy (for Cesarean section); (e) model—(a) plus fetal sex, cesarean section, PE in the current pregnancy and premature rupture of membranes (PROM) (for preterm birth); (f) model—(a) plus prior hypertension (for IUGR); (g) model—(a) plus fetal sex, maternal height, and PE, and GDM in the current pregnancy (for birth weight in percentiles); and (h) model—(a) plus fetal sex, maternal height, and PE and GDM in the current pregnancy, as well as gestational age at birth (for birth weight in grams).

The risk associated with maternal age was assessed in the whole cohort and individual pre-pregnancy BMI subgroups (underweight, norm, overweight, obesity, and BMI ≥25 kg/m^2^).

For a better illustration of the shape and size for the impact of age on risk of pregnancy outcomes results are also shown in the charts. Three types of charts were used. The first is a risk profile chart smoothed by the LOWESS method. The second is a graph, that present the odds ratio and 95% confidence interval obtained in the models. The third (in order) is a bar chart. To better illustrate the odds ratio, the y-axis of both graphs was logarithmized. 

## 3. Results

### 3.1. General Characteristics of Older Women

Table 1 shows the basic characteristics of the participants ≥35 and <35 years of age. In the entire cohort, 52.5% of the women were ≥35 years of age at conception, and the median age of the older women was 37 (36–39). The women aged ≥35 years (vs. <35 years) displayed statistically significantly more frequently pre-pregnancy obesity (12.9% vs. 8.3%) or were overweight (21.5% vs. 16.2%), and used multivitamins (and microelements) during pregnancy statistically significantly less frequently (53% vs. 63%). The older women were also statistically significantly more often poorly educated (11.7% vs. 5.7%) and more often reported a lower financial level (30.1% vs. 28.7%).

In the women aged ≥35 years vs. <35 years (Table 1) we found a statistically significantly higher percentage of preterm deliveries <37th week (9.0% vs. 5.1%) and cesarean sections (regardless of indications) (47.2% vs. 36.0%). Older mothers had a higher percentage of low birth weight (LBW, <2500 g) (8.6% vs. 4.4%) and a higher percentage of macrosomia >4000 g (12.1% vs. 9%). They were more likely to develop isolated gestational hypertension (GH) and gestational diabetes mellitus (GDM-1) (15% and 18.2%, respectively) than the younger ones (9.5% and 8.8%, respectively).

### 3.2. Effects of Maternal Age on the Adverse Pregnancy Results

Table S1 presents maternal age categories among the women developing adverse pregnancy outcomes. In the entire cohort, the most numerous group was aged 35–39 (*n* = 403, 44.2%), and the youngest women (18–24 years old) were the smallest group (*n* = 40, 4.4%).

**Figure 1:**
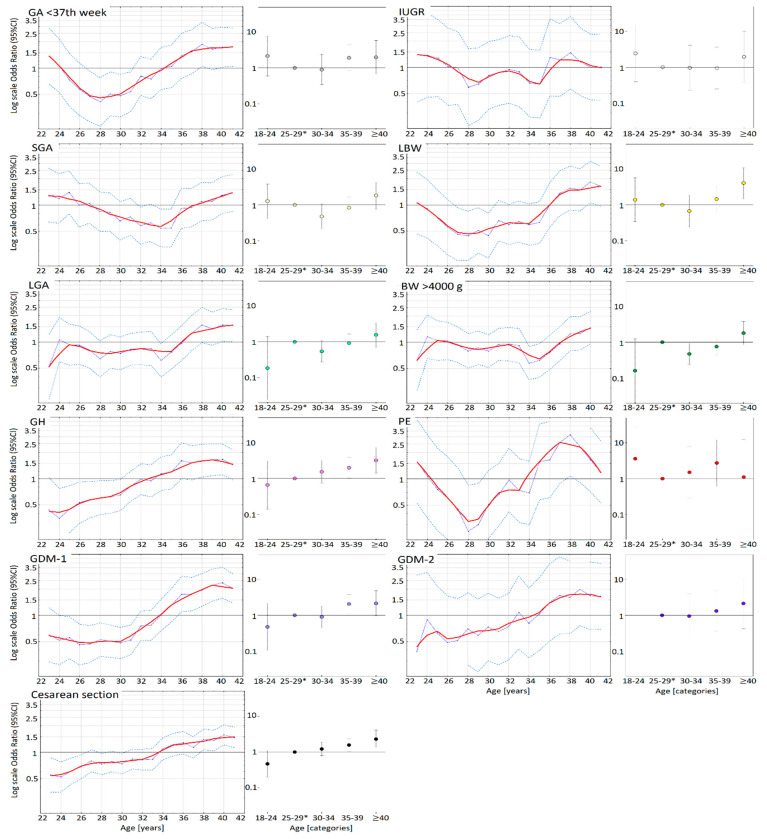
Odds ratios (ORs) of pregnancy outcomes for the mother’s age. The graphs on the left show the odds ratios (and 95% confidence intervals) (blue points), and the risk curve (the red line drawn using the Lowess method) against all maternal age values. The whisker charts (on the right) show the odds ratios (colored dots) and 95% confidence intervals (gray whiskers) for different maternal age categories with regard to the 25–29 [*] years category.

Figure 1 shows the odds ratios of pregnancy outcomes for maternal age. Both younger and older age (Figure 1) was associated with higher odds ratios of PE, IUGR, premature birth <37th week, SGA, and LBW (U-shaped profile). The odds ratios of GH, GDM, and cesarean section increased “linearly” with maternal age. Odds ratios of LGA and macrosomia (>4000 g) increased (after a plateau phase) from the age of 36–37 years on. The maternal threshold age above which the risk of GH, PE, GDM, cesarean section and premature birth increased was approximately 33–34 years, and was lower than the threshold risk of IUGR, LBW, SGA, LGA, and macrosomia >4000 g (approximately 36–37 years).

Table 2 shows the odds ratios of adverse pregnancy outcomes for maternal age with regard to the age range of 25–29 years. This table highlights the U-shaped results; the odds ratios of PE, IUGR, and delivery <37th week were higher both in the youngest and oldest mothers, and the results were maintained after the adjustment, although they remained statistically insignificant. The younger-age relationship with SGA and LBW did not persist after the adjustment. The lowest maternal age (18–24 years) was associated with the lowest odds ratios of the remaining pregnancy outcomes.

Table 3 shows the odds ratios of adverse pregnancy outcomes for maternal age regarding the age range of 18–24 years. This table highlights the results where the odds ratios of pregnancy outcomes increased "linearly" with maternal age (GH, GDM, and cesarean section) or increased to the plateau phase (ages 25–39) and then increased at the age of ≥40 (for LGA and macrosomia, and after adjustment also for SGA and LBW). The youngest age was associated with the highest odds ratios of PE and premature birth (also after adjustment).

Table S2 shows the adjusted odds ratios of adverse pregnancy results for maternal age ≥35 years (compared to <35 years).

### 3.3. The Impact of Maternal BMI on the Effects Associated with Maternal Age

Table S3 shows basic characteristics of the women with excessive BMI (≥25 kg/m^2^). In the cohort 65% (*n* = 593) of the women had normal BMI before pregnancy, 5.2% (*n* = 47) were underweight, 173 19% (*n* = 173) were overweight, and 10.8% (*n* = 98) of the women were obese. The women with overweight and obesity (compared to the women with the normal BMI) had a higher percentage of cesarean sections (45.4% vs. 40.3%), hypertension developed in pregnancy (27.3% and 10.1%,) and gestation diabetes mellitus (21.4% and 13.3%), preterm births <37th week (8.9% vs. 6.2%), and LBW newborns (9.2% vs. 5.4%) as well as macrosomia cases (18.1% vs. 7.4%).

**Figure 2:**
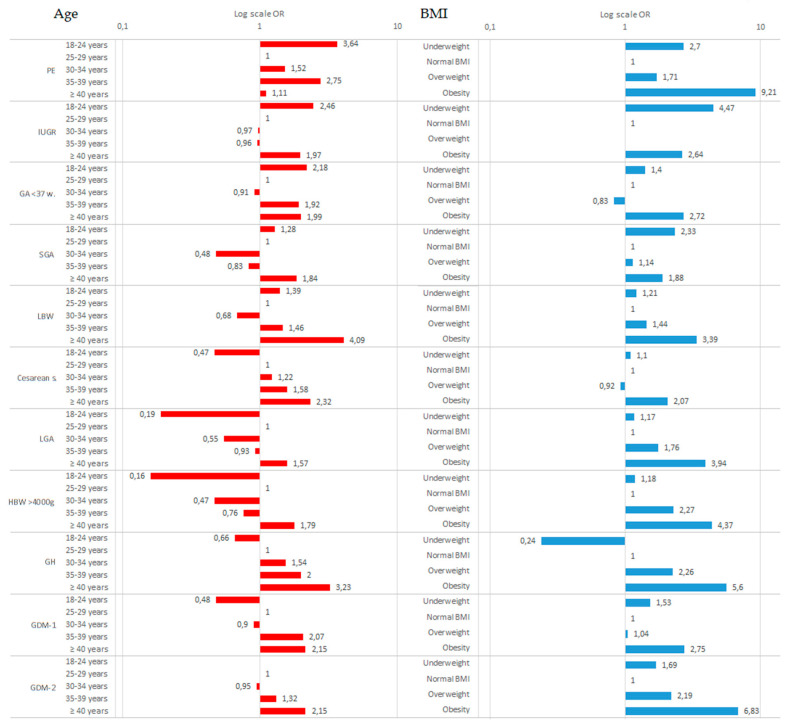
Odds ratios of pregnancy results for (1, Age) maternal age with regard to the age range of 25–29 years, and (2, BMI) maternal weight categories with regard to the Normal BMI.

Figure 2 shows odds ratios of pregnancy results for (1) maternal age with regard to the age range of 25–29 years, and (2) maternal BMI categories with regard to the normal BMI.

(1) This diagram confirms the above presented results indicating that the impact of a pregnant woman’s age on the risk of PE, IGUR, and preterm birth (as well as SGA and LBW) is U shaped. “Linear” growth of risk related to pregnant women’s age can be seen in GH and cesarean section study where the age of 18–24 was associated with the lowest odds ratio (OR = 0.66 and OR = 0.47, respectively). Age 18–24 was strongly preventive of LGA (OR = 0.19), and macrosomia (OR = 0.16).

(2) The impact of inappropriate BMI also reveals clear U shaped relations: at the same time, underweight and obesity (and overweight) involved a higher risk of PE, IUGR, preterm birth, and SGA, as well as diabetes GDM-1 and GDM-2, compared to the women with normal BMI. A “linear” risk increase along with an BMI increase can be seen in GH study, where underweight is related to the lowest odds ratio (OR = 0.24), as compared to the women with normal BMI.

Table 4 and Table S4 show the odds ratios of adverse pregnancy results for maternal age, rated in the whole cohort and subgroups of pre-pregnancy BMI categories. Some values of the odds ratios (OR) had wide confidence intervals confirming low stability of the result which involved the need to repeat the study for larger subgroups. When there were zero cases (in the reference or studied category) “potential” risk was estimated basing on cases/controls relations.

Table 4 shows the effects of obesity, overweight and underweight on the pregnancy outcomes in older mothers (35–39 and 40–45 years old), as compared to those aged 25–29. In the whole cohort, older age of pregnant women was consistent with higher odds ratios for adverse pregnancy outcomes. In obese women, these adverse older age related results were found to be more intense in GH study (OR = 4.29, and OR = 9.33), as well as (though weaker) in birth <37th week study, SGA, LBW, LGA, and macrosomia. In overweight women, these adverse older age related effects were found to be more intense in SGA and LBW study. In underweight women, adverse pregnancy results related to older age were more intense in a study of cesarean section (OR = 7.20). At the same time, underweight was associated with reversal of some negative effects of older age; we found lower odds ratios of GDM-1 diabetes (OR = 0.42).

Table S4 shows the effects of obesity, overweight, and underweight on the pregnancy outcomes in the youngest mothers (18–24 years old) in comparison with 25–29 aged women. The youngest age of mothers was associated with higher odds ratios of PE, IUGR, and preterm birth as well as (though less) with SGA and LBW. The youngest age was associated with lower odds ratios of GH, GDM, cesarean section, LGA and macrosomia (positive effects). In women with BMI ≥25 kg/m^2^, adverse results of the youngest age were found to be more intense in the study of birth <37th week (OR = 5.17) and (though weaker) in PE study. BMI ≥25 kg/m^2^ was associated with a reversal of some. Underweight was associated with a reversal of some positive results of the youngest age; higher odds ratios of LGA and macrosomia for the youngest age were found for underweight women.

## 4. Discussion

In this analysis, we assessed the association of maternal age with unfavorable pregnancy outcomes in a cohort of 912 women with singleton pregnancies who were recruited at the end of the first trimester. We found that maternal age of ≥35 years increased the corrected odds ratios of all tested adverse pregnancy outcomes, in relation to the women of <35 years. However, some pregnancy outcomes were associated with both adolescence and aging: preeclampsia (PE), intrauterine growth restriction (IUGR), and premature birth (<37th week). The lowest odds ratios of PE and IUGR were observed at the age of 25–29, and the lowest odds ratios of premature birth at the age of 30–34.

Findings of this analysis allowed to state that obesity (and overweight) was associated with a higher frequency of all tested adverse pregnancy outcomes, as compared to women with appropriate BMI. U shaped relations were found as well, because underweight also increased the risk of pregnancy adverse effects, with the exception of GH, where underweight was associated with the lowest odds quotient.

The impact of mothers’ obesity, overweight, and underweight on the effects the older age (or the youngest age) was also identified. Obesity and overweight intensified adverse effects of the older age (in relations to GH, preterm birth, SGA, LBW, LGA, and macrosomia) as well as adverse effects of mothers’ youngest age (in relation to PE and birth <37th week). BMI ≥25 kg/m^2^ was associated with a reversal of some positive results of the youngest age in diabetes study. On the other hand, underweight increased adverse effects of the older age in cesarean section study. At the same time, underweight was associated with reversal of negative effects of older age in relations to GDM-1 diabetes. Underweight was also associated with a reversal of positive effects of the youngest age in LGA and macrosomia study.

Our core results (higher risk of pregnancy complications in older mothers, and associations of young age with some pregnancy outcomes) are consistent with several reports in the world literature [4,17,18,19,20,21,28]. However, some studies have found no correlation between maternal age and pregnancy outcomes [2,22]. Differences in clinical methodology, including different age structure of the studied populations, ethnic differences, and different confounding factors could be the cause of the discrepancies between the results. Many studies available in the literature confirm relations between obesity or overweight and adverse pregnancy outcomes or complications [24,25,26].

Our study is a prospective cohort study. At the recruitment, we excluded a priori several factors affecting the risk of pregnancy complications, including chronic diseases (e.g. hypertension and diabetes mellitus) and multiple pregnancy, which may be the cause of a low number of cases of preeclampsia (PE) and gestational diabetes treated with insulin (GDM-2) in our cohort. We also excluded ethnic differences (a risk factor of many adverse health effects) [26]; we recruited Caucasian women from one geographical region of the country, which made the surveyed groups homogenous in terms of the quality of prenatal care. Importantly, our results were maintained after correcting for many confounding variables, including primiparity, pre-pregnancy BMI, weight gain in pregnancy and first trimester smoking, infertility treatment, previous pregnancy hypertension or diabetes, family history of hypertension, and diabetes (among the complications in the mother), as well as the sex of the fetus, the height of the mother, the influence of preeclampsia and diabetes in the current pregnancy, the gestational age of delivery, or the premature rupture of membranes (in the results of the newborn). In many studies the results were not maintained after adjusting for confounding factors [22].

In interpreting our results, we would like to highlight several facts.

In our cohort, a large proportion of women (52.5%) were ≥35 years of age at conception (the median age of the older participants was 37), this was most likely due to the site of the study, at a highly-referenced center where women with risk factors report. The maternal threshold age above which the risk of GH, PE, GDM, caesarean section, and premature birth increased was approximately 33–34 years, and it was lower than the threshold of 35 years assumed in the literature [29]. This threshold risk of developing maternal complications was lower than the threshold risk of IUGR, LBW, SGA, LGA, and macrosomia >4000 g (36–37 years).

The characteristics of our population of older women (Table 1) showed that mothers ≥35 years (compared to those <35 years) were more often obese or overweight, and less frequently used multi-vitamin and micronutrient preparations recommended for pregnant women. They were more often less educated and reported a lower financial status. Literature results confirm that all of these factors may be associated with a higher risk of adverse pregnancy outcomes [30]. In our study, the percentage of women treated for infertility did not differ statistically between older and younger women, and our results for hypertension in pregnancy were adjusted for infertility treatment [31].

Importantly, in our cohort there were 19% overweight women and 10.8% obese ones (Table S2). Women with excessive BMI were more frequently less educated, had a lower financial status, more often smoked, and less often used multi-vitamin supplementation recommended for pregnant women. Excessive gestational weight gain (according to IOM recommendation) in women with BMI ≥25 kg/m^2^ was significantly higher than in women with normal BMI (Table S3).

In our study, in the case of some variables the odds ratio (particularly for subgroups) is based on small quantities. In such cases, for OR > 1, a very wide interval was obtained for an odds ratio, reflecting a small stability of the result, which suggests the need to repeat the study for larger groups. Most of the odds ratios were not statistically significant.

In our study, at the same time, younger and older age was associated with higher adjusted odds ratios of PE, IUGR, and preterm birth <37th week, which is consistent with reports in the literature [17,18,19,32], but the number of PE (*n* = 24) and IUGR (*n* = 21) cases in our the cohort was small. PE and IUGR often coexist, and in their pathogenesis the role of placental ischemia in early pregnancy is taken into account. The risk profiles of PE and preterm birth may also be similar, since the completion of pregnancy (with placental and fetal delivery) remains the primary treatment for preeclampsia (PE) and preterm birth is an important adverse effect of PE [16,17]. The risk profile for SGA was similar (although not identical) to that for IUGR, but we want to emphasize that we defined SGA as <10th percentile (according to percentile grids for gender and gestational age in the study population) and IUGR neonates were pathologically small being both in the <10th percentile and in the newborn group between the 10th and 90th percentile.

Importantly, in obese and overweight women, the adverse older age related effects were found to be more intense in preterm birth study, and SGA study. In women with BMI ≥25 kg/m^2^, adverse outcomes of the youngest age were found to be more intense in study of birth <37th week and (though weaker) in PE study. The subgroup of underweight mothers was small which could have caused that no changes were found in the study of the outcomes. Since placental dysfunctions were found in the study of pregnancy complication pathogenesis, it can be supposed that the analyzed risk factors (advanced maternal age, youngest age, and inappropriate BMI) do contribute to impairment of the placenta functions.

In our study, different maternal age categories were associated with an increased odds ratios of both forms of hypertension developed in pregnancy, isolated gestational hypertension (GH) and preeclampsia (PE), which may emphasize differences in their development mechanisms; the youngest maternal age was associated with higher PE odds ratios, but lower GH odds ratios. However, the common elements of both forms of hypertension were higher odds ratios in older women (Table 3 and Table 4), which in turn indicates the possibility of common pathogenetic mechanisms. Our results are confirmed in the literature [32,33,34]. Based on US databases, Cavazos et al. found a higher risk of PE for the age 11–14, 15–19, 20–24, and ≥40 years, compared to the age 24–29 years. At the same time, the authors found a significant lower adjusted ratio of chances of gestational hypertension (AOR = 0.87) for the age of 20–24 years in relation to 25–29 years [32].

Importantly, in obese women, the adverse effects of older age were increased for GH. In obese women and/or overweight, the adverse effects of the youngest age were found to be more intense in in PE study. Summing up, it was only obesity that increased older age related adverse impact on GH development. This can suggest BMI threshold differences in development of PE and GH or involvement of different mechanisms accompanying obesity and overweight. A small size of the group with underweight women involves the need to verify the results with the use of a larger group.

In our cohort, the risk of gestational hypertension (GH), gestational diabetes mellitus (GM), and caesarean section increased along with increasing maternal age, suggesting that aging influenced these outcomes, and many studies have found the same profile [18]. The rate of cesarean sections in our cohort (without a separate analysis of individual indications) was high (47.2% in women ≥35 years old and 36% in those <35 years old), which is consistent with the literature [4]. It can be expected that with age the cumulative increase in other adverse pregnancy outcomes increased the indications for cesarean section. Interestingly, in our study, older age increased the risk of both macrosomia (>4000 g) and LBW (<2500 g). This effect is similar to the effect of maternal obesity on both high and low birth weight (Supplement Table S2) [16].

Higher odds ratios of GH SGA, LBW, LGA, and macrosomia for an older maternal age and intensification of these effects for macrosomia and LGA in obese women suggest contribution of metabolic disorders in pathogenesis of these outcomes and among obese and older women. Reversal of the effects connected with development of diabetes in underweight women can imply reverse mechanisms of underweight vs. obesity/overweight), though it requires further studies with the use of a larger control group.

The mechanisms of the connections between the older mothers’ age (>35 years) and unfavorable pregnancy outcomes are not clearly explained. The etiology of adverse pregnancy results and complications is multifactorial, and not fully understood, but it includes a significant role of placental ischemia, increased oxidative stress and increased inflammation, as well as endothelial damage [3,14].

Aging is associated with increased oxidative stress and endothelial damage [7,8,9]. The association of older age with a higher risk of PE, FGR, stillbirth and placental ablution may suggest a relation of older age with placental dysfunction [3]. Higher age is associated with reduced beta-cell (pancreatic) function and impaired carbohydrate metabolism as well as insulin resistance [21,35]. All these changes (factors) may increase the risk of hypertension and diabetes in pregnancy. Maternal metabolic disturbances, obesity, diabetes, and hypertension all contribute to higher odds ratios of neonatal macrosomia and/or LBW (LGA and SGA), and are likely an important link between older age (>35 years) and these neonatal outcomes [16]. On the other hand, it is thought that both physiological and organ immaturity is the main factor responsible for the increased risk of some adverse events in pregnancy at an early age. There are suspicions that insulin resistance associated with puberty may be an element that connects the young age of women with preeclampsia [36,37]. The incidence of insulin resistance increases again in older patients and in these age categories there occurs another increase in the risk of preeclampsia [38]. 

Obesity (which in our study exacerbated the effects of age) has been associated with increased oxidative stress and inflammation, as well as with significant changes in the level of chemical compounds with antioxidant properties [11,27]. Relations between obesity and higher risk of preeclampsia, fetal growth restriction, or preterm birth can suggest that obesity is the cause of placental dysfunction [24]. Obesity is associated with an excessive quantity of adipose tissue and released adipokines/cytokines, whose disturbance leads to chronic inflammation and many other metabolic disorders including insulin resistance [39]. Inflammation accompanying obesity, oxidation stress, malfunctioning of numerous neurohormonal, and epigenetic changes have been identified as factors that affect the functions of placenta by changing its ability to nutrient transport [24,39]. Animal models showed relations between obesity and placental blood flow reduction, which can account for higher PE and IUGR risk [24]. At the same time, a higher level of leptin and insulin in obese mothers is related to more intense placental transport of glucose and amino acids to the fetus and its larger mass [24,39]. The relations between the mother’s obesity and preterm birth is considered to be inconclusive and controversial. It is suggested that inflammation that accompany obesity (or advanced maternal age, AMA, >35 years) are preterm birth risk factors [24,40]. The relations between obesity and higher risk of cesarean section (for any reason) can be affected by outcomes such as preeclampsia, diabetes, or macrosomia. Increased inflammation status accompanying obesity which can disturb “the balance of inflammatory factors” and cascade functions of many hormones needed for an appropriate birth, is likely a factor of cesarian section risk [41].

Being underweight can also be a risk factor of adverse pregnancy results because adequate calories and nutrients are needed for the proper development of the fetus. Low level or deficiency of micronutrients (vitamins and elements) with antioxidant properties can increase inflammation and oxidative stress. Indirect effect of other factors, including smoking and other stimulants as well as improper diet or/and coexisting diseases, are also to be taken into account [42,43,44]. However, it has also been found that low calories and low glycaemia can be associated with low levels of oxidative stress [7,8,9], which may explain the reduced effects of more advanced age in our underweight women.

Clinical implications of our results involve the need to optimize body weight through improvement in the life style of women prior to pregnancy [26]. Intervention studies of women prior to pregnancy confirmed a decrease in the risk of adverse pregnancy outcomes after implementation of appropriate diet and physical activity prior to pregnancy. While intervention studies carried out during pregnancies did not reveal statistically significant improvement in pregnancy outcomes [24]. It is suggested that metabolism accompanying obesity (including insulin resistance and hyperinsulinemia) can have an influence on placental gene expression prior to an intervention to be performed which impairs the effects of interventions implemented during pregnancy. It is also thought that interventions during a pregnancy should be increased in the case of obese women [24]. According to the recommendations of the Institute of Medicine (IOM), gestational weight gain (GWG) should be adequate to the mother’s weight prior to pregnancy: 12.5–18 kg, 11.5–16 kg, 7–11.5 kg, and 5–9 kg, for women with underweight, normal weight, overweight, and obesity, respectively. 

Increasing obesity of the society needs to be prevented by implementation of healthy lifestyle of childbearing age women. The study results imply that interventions involving life style improvement (appropriate diet and physical activity) should be aimed at weight reduction prior to pregnancy and between pregnancies. Research suggest that socio-economic indicators should become an important aim for the interventions. Lower levels of education or financial status are associated with elements of unhealthy lifestyle [24,26].

### Limitations and Advantages

The strength of this study was the prospective cohort study model we applied. One strong point was the exclusion of high-risk factors such as pre-pregnancy chronic diseases. Other advantages include the assessment of individual age categories separately in the risk of preeclampsia (PE) and gestational hypertension (GH). One other strength was the calculation of the odds ratios after considering the confounding variables, but we realize that there is a possibility of other confounding variables influencing the results. Additional analyses after the division into subgroups of different BMI categories are yet another advantage of our study.

A limitation, on the other hand, is the small number of women in the age category of 18–24 years and ≥40 years. The limited number of cases of preeclampsia and IUGR is another limitation. The participants of the study reported some anthropometric data (pre-pregnancy weight) themselves.

## 5. Conclusions

Maternal age of ≥35 years increased the odds ratios of all adverse pregnancy outcomes tested, in relation to the age of <35 years. However, the odds ratios of some pregnancy outcomes (preeclampsia, IUGR, and prematurity) were associated with both younger and older age. Importantly, different maternal age categories were associated with increased odds ratios of both forms of hypertension developed in pregnancy, gestational hypertension, and preeclampsia. Importantly, as early as the age of 33, the risk of complications for the mother increased, and it was lower than the threshold of 35 years assumed in the literature. 

In this study, obesity and overweight intensified the older age related pregnancy results (in relations to GH, preterm birth, SGA, LBW, LGA, and macrosomia) as well as adverse effects of mothers’ youngest age (in relation to PE an birth <37th week). BMI ≥25 kg/m^2^ was associated with a reversal of some positive results of the youngest age (in diabetes study). Underweight was also associated with a reversal of positive effects of the youngest age in LGA and macrosomia study.

Our results suggest that mothers aged ≥35 years should avoid obesity, overweight, and underweight. Increasing obesity of the society needs to be prevented by implementation of healthy lifestyle of childbearing age women.

Our results should encourage further research in a larger sample size.

## Figures and Tables

**Table 1:** Basic characteristics of the participants aged <35 years and ≥35 years.

Maternal Characteristics/Pregnancy Outcomes	Women <35 Years (*n* = 433)	Women ≥35 Years (*n* = 479)	
	Median (IQ Range)/*n*(%)	Median (IQ Range)/*n*(%)	*p* *
Maternal age (years)	30 (28–32)	37 (36–39)	<0.001
Primiparity	244 (56.4%)	138 (28.8%)	<0.001
Treatment of infertility	14 (3.2%)	26 (5.4%)	0.106
Pre-pregnancy BMI (kg/m^2^)	22.1 (20.3–24.9)	23.6 (21.1–27.2)	<0.001
BMI categories			<0.001
Obesity	36 (8.3%)	62 (12.9%)	
Overweight	70 (16.2%)	103 (21.5%)	
Normal BMI	295 (68.1%)	299 (62.4%)	
Underweight	32 (7.4%)	15 (3.1%)	
Gestational weight gain (kg)	14 (10–17)	13 (10–17)	0.010
Smoking before pregnancy	84 (19.4%)	84 (17.5%)	0.469
Smoking in I trimester	29 (6.7%)	28 (5.8%)	0.596
Multivitamins in pregnancy	273 (63%)	254 (53%)	0.002
Education <12 years #	22 (5.7%)	48 (11.7%)	0.003
Lower financial status #	62 (23.8%)	69 (30%)	0.125
Fetal sex, son	220 (50.8%)	253 (52.8%)	0.544
Gestational age	39 (38–40)	39 (38–40)	<0.001
Birth <37th week	22 (5.1%)	43 (9.0%)	0.022
IUGR	10 (2.3%)	11 (2.3%)	0.991
Birth weight	3410 (3090–3700)	3400 (3065–3745)	0.947
Birth weight in percentiles			0.460
<10th percentile	30 (6.9%)	42 (8.8%)	
10–90 percentile	365 (84.3%)	376 (78.5%)	
>90th percentile	38 (8.8%)	61 (12.7%)	
Birth weight in grams			0.696
<2500 g	19 (4.4%)	41 (8.6%)	
2500–4000 g	375 (86.6%)	380 (79.3%)	
>4000 g	39 (9.0%)	58 (12.1%)	
APGAR in 5th minute <7	0 (0%)	2 (0.4%)	-
Cesarean section	156 (36.0%)	226 (47.2%)	0.001
GH	41 (9.5%)	72 (15.0%)	0.011
PE	9 (2.1%)	15 (3.1%)	0.321
GDM-1	38 (8.8%)	87 (18.2%)	<0.001
GDM-2	8 (1.8%)	13 (2.7%)	0.384

* The Mann–Whitney U test was used for comparisons of continuous variables, the Cochran–Armitage test was used to detect a trend, and for binomial categories the Pearson chi-square test (or Fisher exact test when Cochran assumption was not met) was used (*p*-value < 0.05 was assumed to be significant); # For available data. Underweight: BMI <18.5 kg/m^2^; normal BMI: 18.5–24.9 kg/m^2^; overweight: BMI 25–29.9 kg/m^2^; obesity: BMI ≥30 kg/m^2^; IUGR: intrauterine growth restriction; the APGAR score evaluates an appearance, pulse, grimace, activity, and respiration; GH: gestational hypertension; PE: preeclampsia; GDM: gestational diabetes mellitus (treated with a diet (-1); with insulin therapy (-2)).

**Table 2:** The odds ratios of adverse pregnancy outcomes for maternal age with regard to the age range of 25–29 years.

Odds Ratios of Pregnancy Results for Maternal Age with Regard to the Age Range of 25–29 Years					
Outcomes	18–24 Years OR (95% CI); *p* AOR (95% CI); *p*	25–29 Years OR (95% CI); *p* AOR (95% CI); *p*	30–34 Years OR (95% CI); *p* AOR (95% CI); *p*	35–39 Years OR (95% CI); *p* AOR (95% CI); *p*	≥40 Years OR (95% CI); *p* AOR (95% CI); *p*
PE	3.64 (0.5–26.74); 0.204	1	1.52 (0.29–7.93); 0.622	2.75 (0.60–12.3); 0.184	1.11 (0.1–12.49); 0.933
	3.48 (0.5–27.14); 0.234	1	1.50 (0.27–8.51); 0.646	2.21 (0.45–10.9); 0.329	0.64 (0.05–8.79); 0.740
IUGR	2.46 (0.4–15.23); 0.334	1	0.97 (0.23–4.13); 0.970	0.96 (0.25–3.65); 0.946	1.97 (0.39–10.0); 0.413
	1.51 (0.2–10.25); 0.673	1	1.15 (0.27–4.99); 0.849	1.30 (0.33–5.23); 0.708	2.40 (0.43–13.43); 0.319
GA <37th w.	2.18 (0.6–7.84); 0.235	1	0.91 (0.34–2.4); 0.839	1.92 (0.84–4.42); 0.125	1.99 (0.67–5.89); 0.216
	1.67 (0.4–7.13); 0.491	1	0.76 (0.27–2.13); 0.606	1.39 (0.56–3.44); 0.479	1.45 (0.45–4.72); 0.539
SGA	1.28 (0.43–3.84); 0.662	1	0.48 (0.21–1.08); 0.077	0.83 (0.42–1.65); 0.596	1.84 (0.77–4.38); 0.169
	0.63 (0.19–2.14); 0.463	1	0.47 (0.20–1.11); 0.085	0.83 (0.39–1.75); 0.622	1.64 (0.62–4.32); 0.317
LBW	1.39 (0.34–5.67); 0.643	1	0.68 (0.25–1.87); 0.451	1.46 (0.63–3.43); 0.38	4.09 (1.52–11.0); 0.005
	0.22 (0.02–2.66); 0.232	1	0.52 (0.13–2.17); 0.371	0.84 (0.24–2.89); 0.783	3.40 (0.81–14.36); 0.096
Cesarean .s	0.47 (0.20–1.1); 0.081	1	1.22 (0.8–1.87); 0.361	1.58 (1.06–2.40); 0.023	2.32 (1.32–4.10); 0.004
	0.38 (0.15–0.93); 0.034	1	1.31 (0.85–2.04); 0.225	1.71 (1.12–2.61); 0.013	2.62 (1.44–4.76); 0.002
LGA	0.19 (0.02–1.43); 0.106	1	0.55 (0.28–1.10); 0.084	0.93 (0.52–1.7); 0.807	1.57 (0.72–3.44); 0.261
	0.21 (0.03–1.81); 0.157	1	0.51 (0.25–1.04); 0.065	0.66 (0.35–1.26); 0.209	0.97 (0.41–2.27); 0.935
HBW > 4000 g	0.16 (0.02–1.25); 0.081	1	0.47 (0.24–0.90); 0.030	0.76 (0.43–1.4); 0.346	1.79 (0.85–3.78); 0.127
	0.16 (0.02–1.36); 0.094	1	0.50 (0.24–1.04); 0.062	0.69 (0.36–1.32); 0.259	1.45 (0.62–3.41); 0.394
GH	0.66 (0.14–3.12); 0.602	1	1.54 (0.74–3.21); 0.244	2.00 (1.0–3.94); 0.044	3.23 (1.41–7.38); 0.005
	0.54 (0.11–2.69); 0.450	1	1.84 (0.82–4.11); 0.137	2.34 (1.10–5.01); 0.028	3.09 (1.21–7.91); 0.018
GDM-1	0.48 (0.10–2.20); 0.342	1	0.90 (0.44–1.82); 0.767	2.07 (1.10–3.80); 0.019	2.15 (0.97–4.80); 0.061
	0.39 (0.08–1.82); 0.228	1	1.11 (0.53–2.29); 0.789	2.55 (1.33–4.9); 0.005	2.72 (1.15–6.42); 0.023
GDM-2	NC	1	0.95 (0.22–4.06); 0.949	1.32 (0.36–4.9); 0.675	2.15 (0.42–11.0); 0.357
	NC	1	0.48 (0.09–2.55); 0.385	0.72 (0.16–3.19); 0.670	0.96 (0.14–6.57); 0.969

OR and AOR: odds ratios and adjusted odds ratios (with 95% confidence intervals) were examined in uni- and multi-dimensional logistic regression, respectively (*p*-value < 0.05 was assumed to be significant), and the confounding variables in multi-dimensional models are described in the Statistical Analysis section. PE: preeclampsia; IUGR: intrauterine growth restriction; GA: gestational age at childbirth; SGA: small-for-gestational age birth weight; LBW: low birth weight; Cesarean s.: Cesarean section; LGA: large-for-gestational age birth weight; HBW: high birth weight; GH: gestational hypertension; GDM: gestational diabetes mellitus (treated with a diet (-1) and with insulin therapy (-2)). NC: no case.

**Table 3:** The odds ratios of adverse pregnancy outcomes for maternal age with regard to the age range of 18–25 years.

Odds Ratios of Pregnancy Results for Maternal Age with Regard to the Age Range of 18–24 Years					
Outcomes	18–24 Years OR (95% CI); *p* AOR (95% CI); *p*	25–29 Years OR (95% CI); *p* AOR (95% CI); *p*	30–34 Years OR (95% CI); *p* AOR (95% CI); *p*	35–39 Years OR (95% CI); *p* AOR (95% CI); *p*	≥40 Years OR (95% CI); *p* AOR (95% CI); *p*
PE	1	0.28 (0.04–2.02); 0.204	0.42 (0.08–2.23); 0.306	0.76 (0.17–3.46); 0.719	0.31 (0.03–3.49); 0.339
	1	0.29 (0.04–2.24); 0.234	0.43 (0.07–2.58); 0.357	0.64 (0.12–3.38); 0.595	0.18 (0.01–2.51); 0.205
IUGR	1	0.41 (0.07–2.53); 0.334	0.40 (0.07–2.11); 0.278	0.39 (0.08–1.9); 0.243	0.80 (0.13–5.02); 0.814
	1	0.66 (0.10–4.50); 0.673	0.76 (0.13–4.64); 0.769	0.86 (0.15–5.07); 0.871	1.59 (0.21–11.86); 0.651
GA <37 w.	1	0.46 (0.13–1.66); 0.235	0.42 (0.13–1.38); 0.151	0.88 (0.3–2.62); 0.822	0.91 (0.25–3.33); 0.890
	1	0.60 (0.14–2.57); 0.491	0.46 (0.11–1.83); 0.270	0.83 (0.22–3.12); 0.786	0.87 (0.19–3.93); 0.855
SGA	1	0.78 (0.26–2.35); 0.662	0.37 (0.12–1.13); 0.081	0.65 (0.24–1.78); 0.404	1.44 (0.46–4.51); 0.533
	1	1.58 (0.47–5.33); 0.463	0.75 (0.22–2.56); 0.641	1.31 (0.4–4.25); 0.655	2.59 (0.69–9.77); 0.161
LBW	1	0.72 (0.18–2.92); 0.643	0.49 (0.13–1.88); 0.297	1.05 (0.31–3.62); 0.937	2.94 (0.77–11.2); 0.114
	1	4.63 (0.38–57.0); 0.232	2.41 (0.2–29.71); 0.492	3.89 (0.35–42.9); 0.268	15.73 (1.3–196.9); 0.033
Cesarean s.	1	2.13 (0.91–4.97); 0.081	2.60 (1.15–5.87); 0.022	3.36 (1.51–7.47); 0.003	4.94 (2.02–12.1); <0.001
	1	2.67 (1.08–6.61); 0.034	3.50 (1.44–8.5); 0.006	4.55 (1.89–11.0); 0.001	6.97 (2.63–18.5); <0.001
LGA	1	5.42 (0.7–42.06); 0.106	2.96 (0.38–22.9); 0.297	5.04 (0.67–37.7); 0.115	8.50 (1.06–68.0); 0.044
	1	4.69 (0.55–40.0); 0.157	2.39 (0.28–20.2); 0.424	3.12 (0.38–25.6); 0.290	4.53 (0.51–40.0); 0.174
HBW > 4000 g	1	6.15 (0.8–47.46); 0.081	2.92 (0.38–22.5); 0.304	4.68 (0.63–35.0); 0.133	11.02 (1.39–87.3); 0.023
	1	6.17 (0.73–52.0); 0.094	3.08 (0.37–25.9); 0.301	4.23 (0.52–34.6); 0.179	8.95 (1.02–78.4); 0.048
GH	1	1.51 (0.32–7.13); 0.602	2.33 (0.53–10.2); 0.261	3.03 (0.71–12.9); 0.135	4.88 (1.06–22.5); 0.042
	1	1.86 (0.37–9.33); 0.450	3.42 (0.73–16.1); 0.119	4.36 (0.94–20.1); 0.059	5.76 (1.14–29.0); 0.034
GDM-1	1	2.09 (0.46–9.63); 0.342	1.88 (0.43–8.34); 0.405	4.33 (1.02–18.4); 0.047	4.51 (0.97–21.0); 0.055
	1	2.60 (0.55–12.3); 0.228	2.87 (0.62–13.2); 0.176	6.64 (1.49–29.6); 0.013	7.06 (1.43–34.7); 0.016
GDM-2	NC	-	-	-	-
	NC	-	-	-	-

OR and AOR: odds ratios and adjusted odds ratios (with 95% confidence intervals) were examined in uni- and multi-dimensional logistic regression, respectively (*p*-value < 0.05 was assumed to be significant), and the confounding variables in multi-dimensional models are described in the Statistical Analysis section. PE: preeclampsia; IUGR: intrauterine growth restriction; GA: gestational age at childbirth; SGA: small-for-gestational age birth weight; LBW: low birth weight; Cesarean s.: Cesarean section; LGA: large-for-gestational age birth weight; HBW: high birth weight; GH: gestational hypertension; GDM: gestational diabetes mellitus (treated with a diet (-1) and with insulin therapy (-2)). NC: no case.

**Table 4:** The odds ratios of adverse pregnancy results for maternal age ≥35 years (with regard to the age range of 25–29 years), rated in the whole cohort and subgroups of pre-pregnancy BMI categories.

Age (Years)	25–29 Years (*n* = 144)		35–39 Years (*n* = 403)		40–45 Years (*n* = 76)
Outcomes/ BMI categories	Cases/Controls	Cases/Controls	OR(95%CI); *p* *	Cases/Controls	OR (95%CI); *p* *
**PE**					
Whole cohort	2/131	14/333	2.75 (0.60–12.3); 0.184	1/59	1.11 (0.1–12.49); 0.933
Underweight	0/10	0/14		0/1	
Normal BMI	1/94	6/224	2.52 (0.3–21.2); 0.396	1/36	2.61 (0.16–42.87); 0.501
Overweight	0/19	2/67		0/16	
Obesity	1/8	6/28	1.71 (0.18–16.4); 0.640	0/6	
BMI ≥25 kg/m^2^	1/27	8/95	2.27 (0.27–18.99); 0,448	0/22	
**IUGR**					
Whole cohort	3/140	8/391	0.96 (0.25–3.65); 0.946	3/71	1.97 (0.39–10.0); 0.413
Underweight	1/9	0/14		0/1	
Normal BMI	2/99	4/252	0.79 (0.14–4.36); 0.783	3/37	4.01 (0.65–24.99); 0.136
Overweight	0/22	0/81		0/20	
Obesity	0/10	4/44		0/13	
BMI ≥25 kg/m^2^	0/32	4/125		0/33	
**GA <37th w.**					
Whole cohort	7/137	36/367	1.92 (0.84–4.42); 0.125	7/69	1.99 (0.67–5.89); 0.216
Underweight	0/10	1/13		0/1	
Normal BMI	6/96	19/238	1.28 (0.5–3.3); 0.613	4/38	1.68 (0.45–6.3); 0.439
Overweight	0/22	5/78		2/18	
Obesity	1/9	11/38	2.61 (0.3–22.87); 0.388	1/12	0.75 (0.04–13.7); 0.846
BMI ≥25 kg/m^2^	1/31	16/116	4.28 (0.55–33.51); 0.167	3/30	3.10 (0.31–31.5); 0.339
**SGA**					
Whole cohort	13/113	31/324	0.83 (0.42–1.65); 0.596	11/52	1.84 (0.77–4.38); 0.169
Underweight	3/6	0/13		0/1	
Normal BMI	8/84	17/216	0.83 (0.34–1.99); 0.67	8/27	3.11 (1.07–9.09); 0.038
Overweight	1/16	9/66	2.18 (0.26–18.49); 0.474	2/15	2.13 (0.18–26.03); 0.553
Obesity	1/7	5/29	1.21 (0.12–12.04); 0.873	1/9	0.78 (0.04–14.75); 0.867
BMI ≥25 kg/m^2^	2/23	14/95	1.70 (0.36–7.99); 0.505	3/24	1.44 (0.22–9.41); 0.705
**LBW**					
Whole cohort	7/117	29/331	1.46 (0.63–3.43); 0.38	12/49	4.09 (1.52–11.0); 0.005
Underweight	0/9	1/12		0/1	
Normal BMI	5/86	12/228	0.91 (0.31–2.65); 0.856	8/26	5.29 (1.59–17.58); 0.007
Overweight	1/15	7/65	1.62 (0.19–14.14); 0.665	3/13	3.46 (0.32–37.47); 0.307
Obesity	1/7	9/26	2.42 (0.26–22.49); 0.436	1/9	0.78 (0.04–14.75); 0.867
BMI ≥25 kg/m^2^	2/22	16/91	1.93 (0.41–9.04); 0.402	4/22	2.00 (0.33–12.07); 0.450
**Cesarean s.**					
Whole cohort	50/94	184/219	1.58 (1.06–2.40); 0.023	42/34	2.32 (1.32–4.10); 0.004
Underweight	2/8	9/5	7.20 (1.08–47.96); 0.041	0/1	
Normal BMI	33/69	116/141	1.72 (1.06–2.79); 0.027	24/18	2.79 (1.33–5.84); 0.007
Overweight	9/13	28/55	0.74 (0.28–1.93); 0.532	8/12	0.96 (0.28–3.31); 0.952
Obesity	6/4	31/18	1.15 (0.29–4.62); 0.846	10/3	2.22 (0.37–13.54); 0.386
BMI ≥25 kg/m^2^	15/17	59/73	0.92 (0.42–1.99); 0.824	18/15	1.36 (0.51–3.61); 0.537
**LGA**					
Whole cohort	18/113	48/324	0.93 (0.52–1.7); 0.807	13/52	1.57 (0.72–3.44); 0.261
Underweight	1/6	1/13	0.46 (0.03–8.69); 0.606	0/1	
Normal BMI	10/84	24/216	0.93 (0.43–2.04); 0.862	7/27	2.18 (0.76–6.28); 0.15
Overweight	5/16	8/66	0.39 (0.11–1.35); 0.136	3/15	0.64 (0.13–3.16); 0.583
Obesity	2/7	15/29	1.81 (0.33–9.82); 0.491	3/9	1.17 (0.15–9.01); 0.882
BMI ≥25 kg/m^2^	7/23	23/95	0.80 (0.3–2.08); 0.641	6/24	0.82 (0.24–2.81); 0.754
**HBW >4000 g**					
Whole cohort	20/117	43/331	0.76 (0.43–1.4); 0.346	15/49	1.79 (0.85–3.78); 0.127
Underweight	1/9	1/12	0.75 (0.04–13.68); 0.846	0/1	
Normal BMI	11/86	17/228	0.58 (0.26–1.3); 0.185	8/26	2.41 (0.88–6.61); 0.089
Overweight	6/15	11/65	0.42 (0.14–1.33); 0.14	4/13	0.77 (0.18–3.34); 0.726
Obesity	2/7	14/26	1.89 (0.34–10.32); 0.465	3/9	1.17 (0.15–9.01); 0.882
BMI ≥25 kg/m^2^	8/22	25/91	0.76 (0.3–1.9); 0.551	7/22	0.88 (0.27–2.83); 0.824
**GH**					
Whole cohort	11/131	56/333	2.00 (1.0–3.94); 0.044	16/59	3.23 (1.41–7.38); 0.005
Underweight	0/10	0/14		0/1	
Normal BMI	7/94	27/224	1.62 (0.68–3.85); 0.275	5/36	1.87 (0.56–6.26); 0.313
Overweight	3/19	14/67	1.32 (0.34–5.09); 0.683	4/16	1.58 (0.31–8.15); 0.582
Obesity	1/8	15/28	4.29 (0.49–37.59); 0.189	7/6	9.33 (0.89–97.62); 0.062
BMI ≥25 kg/m^2^	4/27	29/95	2.06 (0.67–6.38); 0.21	11/22	3.38 (0.94–12.08); 0.062
**GDM-1**					
Whole cohort	14/127	73/320	2.07 (1.10–3.80); 0.019	14/59	2.15 (0.97–4.80); 0.061
Underweight	3/7	2/11	0.42 (0.06–3.21); 0.407	0/1	
Normal BMI	7/93	43/211	2.71 (1.17–6.24); 0.019	7/34	2.74 (0.89–8.37); 0.078
Overweight	2/20	13/67	1.94 (0.4–9.33); 0.408	2/16	1.25 (0.16–9.88); 0.832
Obesity	2/7	15/31	1.69 (0.31–9.16); 0.541	5/8	2.19 (0.32–15.04); 0.426
BMI ≥25 kg/m^2^	4/27	28/98	1.93 (0.62–5.98); 0.255	7/24	1.97 (0.51–7.56); 0.324
**GDM-2**					
Whole cohort	3/127	10/320	1.32 (0.36–4.9); 0.675	3/59	2.15 (0.42–11.0); 0.357
Underweight	0/7	1/11		0/1	
Normal BMI	2/93	3/211	0.66 (0.11–4.02); 0.653	1/34	1.37 (0.12–15.57); 0.801
Overweight	0/20	3/67		2/16	
Obesity	1/7	3/31	0.68 (0.06–7.52); 0.751	0/8	
BMI ≥25 kg/m^2^	1/27	6/98	1.65 (0.19–14.33); 0.648	2/24	2.25 (0.19–26.41); 0.519

* OR: crude odds ratios (with 95% confidence intervals) were examined in the uni-dimensional logistic regression (*p*-value < 0.05 was assumed to be significant). Underweight: BMI < 18.5 kg/m^2^; normal BMI: 18.5–24.9 kg/m^2^; overweight: BMI 25–29.9 kg/m^2^; obesity: BMI ≥30 kg/m^2^; PE: preeclampsia; IUGR: intrauterine growth restriction; GA: gestational age at childbirth; SGA: small-for-gestational age birth weight; LBW: low birth weight; Cesarean s.: Cesarean section; LGA: large-for-gestational age birth weight; HBW: high birth weight; GH: gestational hypertension; GDM: gestational diabetes mellitus (treated with a diet (-1) and with insulin therapy (-2)).

## Supplementary Materials

The following are available online at https://www.mdpi.com/2072-6643/12/9/2838/s1, Table S1: Maternal age categories among the women developing adverse pregnancy outcomes; Table S2: The adjusted odds ratios of adverse pregnancy results for maternal age ≥35 years; Table S3: Basic characteristics of the women with overweight and obesity; Table S4: The odds ratios of adverse pregnancy results for maternal age 18–24 years (with regard to the age range of 25–29 years), rated in the whole cohort and subgroups of pre-pregnancy BMI categories. All authors have read and agreed to the published version of the manuscript.
