# Supplementary material for: The Influence of Maternal BMI on Adverse Pregnancy Outcomes in Older Women
Source: Nutrients. 2020 Sep 16;12(9):2838. doi: 10.3390/nu12092838 (PMC7551140; doi:10.3390/nu12092838)
Supplement: Supplementary file 1 [file nutrients-12-02838-s001.pdf]

nutrients

Supplementary Materials

Table S1. Maternal age categories among the women developing adverse pregnancy outcomes.

Characteristics of maternal age categories in pregnancy results #					
**Outcomes**	**18–24 Years**	**25–29 Years**	**30–34 Years**	**35–39 Years**	**≥ 40 Years**
Total	*n* = 40 (4.4%)	*n* =144 (15.8%)	*n* = 249 (27.3%)	*n* = 403 (44.2%)	*n* = 76 (8.3%)
Whole cohort	40 (4.4%)	144 (15.8%)	249 (27.3%)	403 (44.2%)	76 (8.3%)
PE	2 (8.3%)	2 (8.3%)	5 (20.8%)	14 (58.3%)	1 (4.2%)
IUGR	2 (9.5%)	3 (14.3%)	5 (23.8%)	8 (38.1%)	3 (14.3%)
Birth <37th week	4 (6.2%)	7 (10.8%)	11 (16.9%)	36 (55.4%)	7 (10.8%)
SGA	5 (6.9%)	13 (18.1%)	12 (16.7%)	31 (43.1%)	11 (15.3%)
LBW **	3 (5.0%)	7 (11.7%)	9 (15.0%)	29 (48.3%)	12 (20.0%)
Cesarean s. ***	8 (2.1%)	50 (13.1%)	98 (25.7%)	184 (48.2%)	42 (11%)
LGA *	1 (1.0%)	18 (18.2%)	19 (19.2%)	48 (48.5%)	13 (13.1%)
HBW >4000g *	1 (1.0%)	20 (20.6%)	18 (18.6%)	43 (44.3%)	15 (15.5%)
GH ***	2 (1.8%)	11 (9.7%)	28 (24.8%)	56 (49.6%)	16 (14.2%)
GDM-1 ***	2 (1.6%)	14 (11.2%)	22 (17.6%)	73 (58.4%)	14 (11.2%)
GDM-2	0 (0%)	3 (14.3%)	5 (23.8%)	10 (47.6%)	3 (14.3%)

For each pregnancy result, the Cochran–Armitage test was used to detect a trend of maternal age categories (and *p*-value < 0.05 was assumed to be significant, for comparisons of the trend between cases and controls); * *p*-value was < 0.05; ** *p*-value was < 0.01; *** *p*-value was ≤ 0.001. PE: preeclampsia; IUGR: intrauterine growth restriction; SGA: small-for-gestational age birth weight; LBW: low birth weight; Cesarean s.: Cesarean section; LGA: large-for-gestational age birth weight; HBW: high birth weight; GH: gestational hypertension; GDM: gestational diabetes mellitus (treated with a diet (-1) and with insulin therapy (-2)).

Table S2. The adjusted odds ratios of adverse pregnancy results for maternal age ≥ 35 years.

Adjusted odds ratios of pregnancy results for maternal age ≥ 35 years			
**Pregnancy results**	**< 35 Years**	**≥ 35 Years**	**≥ 35 Years**
	Ref	OR (95% CI)	AOR (95% CI)
PE	1	1.63 (0.7-3.77); 0.254	1.28 (0.51-3.2); 0.604
IUGR	1	1.00 (0.42-2.37); 0.991	1.29 (0.51-3.27); 0.59
Birth < 37th week	1	1.84 (1.08-3.13); 0.024	1.55 (0.85-2.83); 0.152
SGA	1	1.36 (0.83-2.22); 0.220	1.47 (0.85-2.54); 0.167
LBW	1	2.13 (1.21-3.74); 0.008	2.27 (1.25-4.12); 0.007
Cesarean section	1	1.59 (1.22-2.07); 0.001	1.63 (1.22-2.17); 0.001
LGA	1	1.56 (1.01-2.4); 0.043	1.23 (0.78-1.94); 0.374
HBW (> 4000g)	1	1.47 (0.95-2.26); 0.081	1.29 (0.79-2.08); 0.308
GH	1	1.72 (1.14-2.58); 0.010	1.75 (1.09-2.81); 0.021
GDM-1	1	2.34 (1.56-3.51); <0.001	2.60 (1.67-4.06); <0.001
GDM-2	1	1.66 (0.68-4.05); 0.266	1.35 (0.48-3.77); 0.573

OR and AOR: odds ratios and adjusted odds ratios (with 95% confidence intervals) were examined in uni- and multi-dimensional logistic regression, respectively (*p*-value < 0.05 was assumed to be significant), and the confounding variables in multi-dimensional models are described in the Statistical Analysis section. PE: preeclampsia; IUGR: intrauterine growth restriction; SGA: small-for-gestational age birth weight; LBW: low birth weight; HBW: high birth weight; GH: gestational hypertension; GDM: gestational diabetes mellitus (treated with a diet (-1) and with insulin therapy (-2)).

Table S3. Basic characteristics of the women with overweight and obesity.

**Maternal characteristics / Pregnancy outcomes**	**BMI ≥ 25 kg/m²** (***n*** **= 272)**	**Normal BMI** (***n*** **= 593)**	
	**Median (IQ range)/*n*(%)**	**Median (IQ range)/*n*(%)**	***p****
Pre-pregnancy BMI (kg/m²)	28.7 (26.7-31.3)	21.7 (20.3-23.2)	<0.0001
Overweight	173	-	
Obesity ≥30 kg/m²	98	-	
Edema	17 (6.3%)	24 (4%)	0.153
Gestational weight gain GWG categories:			<0.0001
below the range	39 (14.4%)	177 (29.8%)	
in the range	72 (26.6%)	246 (41.5%)	
above the range	160 (59%)	170 (28.7%)	
Maternal age (years)	35 (32-37)	35 (30-37)	0.003
Age categories			
18–24	7 (2.6%)	27 (4.6%)	0.0005
25–29	32 (11.8%)	102 (17.2%)	
30–34	67 (24.7%)	165 (27.8%)	
35–39	132 (48.7%)	257 (43.3%)	
≥ 40	33 (12.2%)	42 (7.1%)	
Primiparity	101 (37.3%)	250 (42.2%)	0.175
Smoking before pregnancy	65 (24%)	90 (15.2%)	0.002
Multivitamins in pregnancy	147 (54.2%)	352 (59.4%)	0.158
Education <12 years #	34 (14.3%)	30 (5.8%)	0.0001
Lower financial status #	67 (41.6%)	58 (19.2%)	<0.0001
Fetal sex. daughter	128 (47.2%)	287 (48.4%)	0.750
Birth < 37th week	24 (8.9%)	37 (6.2%)	0.164
Birth weight in percentiles:			
< 10th percentile	23 (8.5%)	42 (7.1%)	0.0002
10–90 percentile	201 (74.2%)	503 (84.8%)	
> 90th percentile	47 (17.3%)	48 (8.1%)	
Birth weight in grams:			<0.0001

< 2500 g	25 (9.2%)	32 (5.4%)	
2500–4000 g	197 (72.7%)	517 (87.2%)	
> 4000 g	49 (18.1%)	44 (7.4%)	
APGAR in 5th minute < 7	0 (0%)	2 (0.3%)	-
Cesarean section	123 (45.4%)	239 (40.3%)	0.160
PIH	74 (27.3%)	60 (10.1%)	<0.0001
GDM	58 (21.4%)	79 (13.3%)	0.003

* The Mann–Whitney U test was used for comparisons of continuous variables, the Cochran–Armitage test was used to detect a trend, and for binomial categories the Pearson chi-square test (or Fisher exact test when Cochran assumption was not met) was used (*p*-value < 0.05 was assumed to be significant); # For available data. The APGAR score evaluates an appearance, pulse, grimace, activity, and respiration; PIH: pregnancy induced hypertension (preeclampsia and isolated gestational hypertension); GDM: gestational diabetes mellitus (treated with a diet and treated with insulin therapy).**Table S4.** The odds ratios of adverse pregnancy results for maternal age 18– 24 years (with regard to the age range of 25– 29 years), rated in the whole cohort and subgroups of pre-pregnancy BMI categories.

Table S4. The odds ratios of adverse pregnancy results for maternal age 18– 24 years (with regard to the age range of 25– 29 years), rated in the whole cohort and subgroups of pre-pregnancy BMI categories.

**Age**		**18–24 Years (*n* = 40)**	**25–29 Years (*n* = 144)**
**Outcomes/ BMI categories**	**Cases/ Controls**	**OR(95%CI); *p* ***	**Cases/ Controls**
**PE**			
Whole cohort	2/36	3.64 (0.5-26.74); 0.204	2/131
Underweight	1/5		0/10
Normal BMI	0/25		1/94
Overweight	1/4		0/19
Obesity	0/2		1/8
BMI ≥25 kg/m²	1/6	4.50 (0.25-82.57); 0.311	1/27
**IUGR**			
Whole cohort	2/38	2.46 (0.4-15.23); 0.334	3/140
Underweight	1/5	1.80 (0.09-35.42); 0.699	1/9
Normal BMI	1/26	1.90 (0.17-21.82); 0.605	2/99
Overweight	0/5		0/22
Obesity	0/2		0/10
BMI ≥25 kg/m²	0/7		0/32
**GA < 37th w.**			
Whole cohort	4/36	2.18 (0.6-7.84); 0.235	7/137
Underweight	1/5		0/10
Normal BMI	2/25	1.28 (0.24-6.73); 0.771	6/96
Overweight	1/4		0/22
Obesity	0/2		1/9
BMI ≥25 kg/m²	1/6	5.17 (0.28-94.5); 0.268	1/31
**SGA**			
Whole cohort	5/34	1.28 (0.43-3.84); 0.662	13/113
Underweight	1/4	0.50 (0.04-6.68); 0.600	3/6
Normal BMI	4/23	1.83 (0.51-6.61); 0.359	8/84

Overweight	0/5		1/16
Obesity	0/2		1/7
BMI ≥25 kg/m²	0/7		2/23
**LBW**			
Whole cohort	3/36	1.39 (0.34-5.67); 0.643	7/117
Underweight	1/4		0/9
Normal BMI	2/25	1.38 (0.25-7.53); 0.713	5/86
Overweight	0/5		1/15
Obesity	0/2		1/7
BMI ≥25 kg/m²	0/7		2/22
**Cesarean s.**			
Whole cohort	8/32	0.47 (0.2-1.1); 0.081	50/94
Underweight	1/5	0.80 (0.06-11.3); 0.869	2/8
Normal BMI	6/21	0.60 (0.22-1.62); 0.311	33/69
Overweight	1/4	0.36 (0.03-3.79); 0.396	9/13
Obesity	0/2		6/4
BMI ≥25 kg/m²	1/6	0.19 (0.02-1.75); 0.143	15/17
**LGA**			
Whole cohort	1/34	0.19 (0.02-1.43); 0.106	18/113
Underweight	1/4	1.50 (0.07-31.58); 0.794	1/6
Normal BMI	0/23		10/84
Overweight	0/5		5/16
Obesity	0/2		2/7
BMI ≥25 kg/m²	0/7		7/23
**HBW >4000g**			
Whole cohort	1/36	0.16 (0.02-1.25); 0.081	20/117
Underweight	1/4	2.25 (0.11-45.72); 0.598	1/9
Normal BMI	0/25		11/86
Overweight	0/5		6/15
Obesity	0/2		2/7
BMI ≥25 kg/m²	0/7		8/22
**GH**			

Whole cohort	2/36	0.66 (0.14-3.12); 0.602	11/131
Underweight	0/5		0/10
Normal BMI	2/25	1.07 (0.21-5.5); 0.931	7/94
Overweight	0/4		3/19
Obesity	0/2		1/8
BMI ≥25 kg/m²	0/6		4/27
**GDM-1**			
Whole cohort	2/38	0.48 (0.1-2.2); 0.342	14/127
Underweight	0/6		3/7
Normal BMI	1/26	0.51 (0.06-4.34); 0.539	7/93
Overweight	0/5		2/20
Obesity	1/1	3.50 (0.15-84.69); 0.441	2/7
BMI ≥25 kg/m²	1/13	1.13 (0.11-11.95); 0.922	4/27
**GDM-2**			
Whole cohort	0/38		3/127
Underweight	0/6		0/7
Normal BMI	0/26		2/93
Overweight	0/5		0/20
Obesity	0/1		1/7
BMI ≥25 kg/m²	0/6		1/27

* OR: crude odds ratios (with 95% confidence intervals) were examined in the uni-dimensional logistic regression (*p*-value < 0.05 was assumed to be significant). PE: preeclampsia; IUGR: intrauterine growth restriction; GA: gestational age at childbirth; SGA: small-for-gestational age birth weight; LBW: low birth weight; Cesarean s.: Cesarean section; LGA: large-for-gestational age birth weight; HBW: high birth weight; GH: gestational hypertension; GDM: gestational diabetes mellitus (treated with a diet (-1) and with insulin therapy (-2)).
